# Cartas al editor

**Published:** 2020-11-15

**Authors:** David A. Forero-Peña, Natasha A. Camejo-Ávila, Fhabián S. Carrión-Nessi

**Affiliations:** 1 Instituto de Investigación Biomédica y Vacunas Terapéuticas, Ciudad Bolívar, Venezuela; Departamento de Enfermedades Infecciosas del Adulto, Hospital Universitario de Caracas, Caracas, Distrito Capital, Venezuela Hospital Universitario de Maracaibo Hospital Universitario de Caracas CaracasDistrito Capital Venezuela; 2 Instituto de Investigación Biomédica y Vacunas Terapéuticas, Ciudad Bolívar, Venezuela Instituto de Investigación Biomédica y Vacunas Terapéuticas Bolívar Venezuela

Caracas, 19 de octubre de 2020

Señores Comité Editorial Revista *Biomédica* Bogotá, D.C.

Estimados señores:

Hemos leído con interés la reciente publicación de Gregorio-Chaviano, *et al.*^1^, en la que analizan la producción científica sobre la COVID-19 en Latinoamérica, y quisiéramos hacer algunas consideraciones que nos parecen importantes.

En primer lugar, pese a que los autores realizaron un interesante estudio bibliométrico de los documentos sobre la COVID-19 en Latinoamérica, el análisis de la producción científica no necesariamente representa un aporte intelectual tangible a la comprensión de la enfermedad, ya que los estudios bibliométricos sólo informan sobre la cantidad de publicaciones, no sobre su calidad, por lo que son frecuentemente combinados con indicadores de impacto o el juicio de expertos ^2^. Recientemente publicamos una revisión sistemática y análisis bibliométrico de la producción científica sobre la COVID-19 en Latinoamérica, en la que analizamos la calidad de los documentos recuperados, los reclasificamos y describimos su contribución científica real ^3^. Casi para la misma fecha de búsqueda, recuperamos 101 documentos en comparación con los 142 recuperados por Gregorio-Chaviano, *et al.,* probablemente debido a las diferentes bases de datos consultadas y estrategias de búsqueda construidas en los dos estudios. En el nuestro, la mayoría (72/101; 71,3 %) de los documentos recuperados eran comentarios u opiniones, editoriales o perspectivas, recomendaciones clínicas y revisiones narrativas que, si bien representan un «producto científico», no van más allá de publicaciones sin contenido inédito, por lo que incluimos únicamente los documentos que aportaban un conocimiento real: investigaciones en ciencias básicas, reportes y series de casos, estudios transversales, ensayos clínicos y metaanálisis.

Por otra parte, aunque diversos estudios bibliométricos sobre la COVID-19 han analizado el comportamiento de la producción científica en el mundo, consideramos que algunas de esas aproximaciones pueden tener sesgos en el contexto de esta pandemia, pues incluyen documentos sin previa revisión de pares y no evalúan su contenido o calidad. Gregorio-Chaviano, *et al.,* señalan que «la publicación de los artículos en revistas internacionales, en su mayoría de elevada visibilidad e impacto, muestra una tendencia positiva en cuanto a la calidad de las investigaciones» ^1^; sin embargo, se ha señalado que, más que de otros tipos de estudios, son altas las tasas de citación de documentos metodológicos y de protocolos que introducen técnicas y reglamentos referenciados para cada uso, así como revisiones de la literatura, debido a que manejan una amplia bibliografía y su consulta es especialmente útil para los científicos ^2^. Además, el recuento de citas se ve afectado no solo por el idioma, el campo de estudio o el tipo de artículo (investigación original, editorial, revisión, etc.), sino también por otras razones subjetivas ^4^. Amin, *et al.*^5^, consideran que deben discriminarse los «artículos válidos» -aquellos con suficiente información para asegurar las observaciones, repetir las experiencias y evaluar los procesos intelectuales- de los otros tipos de publicaciones, debido a que ello influye en el análisis y en las conclusiones relacionadas con la producción de conocimientos científicos.

Actualmente, una gran cantidad de artículos científicos sobre la COVID-19 se difunden rápidamente en diferentes redes sociales, incluso los de baja calidad, y pocas veces se constatan los resultados. La disputa por «publicar o perecer» está llevando a los autores y a las revistas a someter y aceptar resultados de baja calidad o dudosa credibilidad; por ejemplo, recientemente la prestigiosa revista *Lancet* publicó un estudio sobre el papel de la hidroxicloroquina en el tratamiento de la COVID-19 que posteriormente fue retractado por los propios autores ^6^. En este momento de crisis sanitaria por la COVID-19, es importante que las publicaciones sean de alta calidad y aporten conocimientos verdaderos a la comprensión de la enfermedad.

En conclusión, nosotros encontramos (hasta la fecha de búsqueda) que dos de cada tres artículos publicados en Latinoamérica no aportan conocimiento nuevo en la comprensión del impacto de la COVID-19 en la región. Además, si solo analizamos este tipo de publicaciones, no podríamos estimar realmente el liderazgo científico ni la capacidad de generar proyectos de investigación, sino simplemente el número de artículos publicados.
