# Supplementary material for: Cartas al editor
Source: Biomedica. 2020 Nov 15;40(Suppl 2):205–8. [Article in Spanish] (PMC7676833)
Supplement: Related texts

# Reply: Cartas al editor

Bogotá, 22 de octubre de 2020

Señores Comité Editorial Revista Biomédica

Bogotá, D.C.

Apreciados señores:

Agradecemos a los autores de la carta al editor la lectura de nuestro artículo y los aportes y consideraciones que hicieron sobre él.

Tal como lo exponen los autores de la carta, nuestro artículo no representa aportes intelectuales a la comprensión de la enfermedad, pues se trata de un estudio meramente descriptivo en el que nos limitamos a exponer el comportamiento de la producción bibliográfica en las primeras semanas del año, haciendo énfasis en los autores e instituciones participantes en los estudios, así como en el crecimiento exponencial de la investigación.

En el momento en que escribimos el manuscrito nuestro principal objetivo era saber y describir a quienes estaban participando a nivel regional en la investigación de un tema que, por su impacto médico y social, generaba y genera gran interés. No nos planteamos ir más allá para analizar la estructura del contenido a través de las citas, aspecto que hoy resulta clave dado el volumen de estudios publicados, cuyo análisis aportaría más información sobre los detalles propios de las investigaciones y su impacto real en el manejo de la enfermedad, y ameritaría centrarse en aquellos estudios enfocados al desarrollo de vacunas, por ejemplo.

Cualquier estudio bibliométrico parte de sesgos: desde las bases de datos empleadas, la propia selección de términos y la ecuación de búsqueda, hasta los análisis propiamente dichos. En nuestro estudio utilizamos tres fuentes intentando cubrir una amplia cantidad de información dado el breve periodo analizado, pero dejando por fuera la producción registrada en otras bases de datos y las tipologías documentales que aportan al conocimiento de la enfermedad. Durante este año otros estudios se han centrado en análisis descriptivos, exponiendo el ritmo de crecimiento ^1^, el incremento de la producción, las revistas que concentran las publicaciones, y las tasas de colaboración, entre otros ^2^^-^^4^.

Como lo plantean los colegas en su carta, el síndrome de "publicar o perecer" se expresa en la mayoría de los países e instituciones, debido, entre otros aspectos, al tipo de evaluación de investigadores y universidades, así como a las políticas de incentivos a la investigación. Hasta este momento, por mencionar solo la base de Scopus, hay registrados 11.972 documentos citables en el área médica, cantidad que, sin duda, irá en aumento y aportará información clave para la adopción de decisiones y la gestión de la pandemia, campos en los que la bibliometría y sus métodos desempeñan un papel relevante para conocer las dinámicas de la investigación en un momento dado. Es preciso continuar produciendo estudios bibliométricos descriptivos y de contenido, pues ambos aportan información valiosa sobre las prácticas de investigación. La invitación es a elevar la calidad de dichos estudios y analizar los detalles de la investigación que se publica para brindar herramientas a la comunidad científica y a la sociedad que contribuyan a dejar atrás estos tiempos de pandemia.

Cordialmente,

## Orlando Gregorio-Chaviano

Departamento de Ciencias de la Información, Facultad de Comunicación y Lenguaje, Pontificia Universidad Javeriana, Bogotá, D.C., Colombia

## César H. Limaymanta

Línea de Estadística, Departamento de Ciencias, Universidad Peruana de Ciencias Aplicadas, Lima, Perú

## Evony K. López-Mesa

Coordinación de Servicios al Usuario, Biblioteca, Universidad La Gran Colombia, Bogotá, D.C., Colombia

## References

[1] Torres-Salinas D (2020). Ritmo de crecimiento diario de la producción científica sobre COVID-19. Análisis en bases de datos y repositorios en acceso abierto. EPI.

[2] O'Brien N, Barboza-Palomino M, Ventura-León J, Caycho-Rodríguez T, Sandoval-Díaz JS, López-López W (2020). Nuevo coronavirus (COVID-19). Un análisis bibliométrico. Revista Chilena de Anestesia.

[3] Pascual CT, Torrell-Vallespín S (2020). Análisis bibliométrico de la producción científica latinoamericana y del Caribe sobre COVID-19 en PUBMED. Rev Cubana Pediatr.

[4] Núñez RO (2020). Análisis métrico de la producción científica sobre COVID-19 en Scopus (2019-abril 2020). Revista Cubana de Información en Ciencias de la Salud.
